# Measurement-based care vs. standard care for major depressive disorder in Pakistan: protocol for a randomized control trial

**DOI:** 10.1192/j.eurpsy.2023.1770

**Published:** 2023-07-19

**Authors:** I. Husain, M. Umer, Z. Nigah, T. Kiran, A. Bukhsh, M. Ansari, M. R. Bhatia, O. Husain, H. Naqvi, A. Qadir, M. Saqib, A. H. Rajput, M. A. Zeb, S. A. Khan, K. M. S. Siddiqui, S. Sherzad, B. Mulsant, N. Chaudhry, I. Chaudhry, N. Husain

**Affiliations:** 1 Centre for Addiction and Mental Health, Toronto, Canada; 2 Ishrat Husain Pakistan Institute of Living and Learning, Karachi; 3 Liaquat University of Medical & Health Sciences, Cowasjee Jehangir Institute of Psychiatry, Hyderbabad; 4 Peoples University of Medical and Health Sciences, Nawabshah; 5 Dow University of Health Sciences, Karachi; 6 Baluchistan Institute of Psychiatry and Behavioral Sciences, Quetta; 7 North West General Hospital, Peshawar; 8 National Psychiatric Hospital, Multan; 9 Ziauddin University, Karachi, Pakistan; 10 Division of Psychology and Mental Health, School of Health Sciences, University of Manchester, Manchester, United Kingdom

## Abstract

**Introduction:**

Low and middle-income countries (LMICs) hold the majority of disease burden attributed to major depressive disorder (MDD). Despite this, there remains a substantial gap for access to evidence-based treatments for MDD in LMICs like Pakistan. Measurement-based care (MBC) incorporates systematic administration of validated outcome measures to guide treatment decision making and is considered a low-cost approach to optimise better clinical outcomes for individuals with MDD but there is a paucity of evidence on the efficacy of MBC in LMICs.

**Objectives:**

This protocol highlights a randomized trial which will include Pakistani outpatients with moderate to severe major depression.

**Methods:**

Participants will be randomised to either MBC (guided by schedule), or standard treatment (guided by clinicians’ judgement), and will be prescribed with paroxetine (10–60mg/day) or mirtazapine (7.5–45mg/day) for 24 weeks. Outcomes will be evaluated by raters blind to study protocol and treatment.

**Results:**

National Bioethics Committee (NBC) of Pakistan has given full ethics approval. The trial is being conducted and reported as per recommendation of the CONSORT statement for RCTs.

**Conclusions:**

With increasing evidence from high-income settings supporting the effectiveness of MBC for MDD, it is now necessary to explore its feasibility, utility. and efficacy in low-resource settings. The results of the proposed trial could inform the development of a low-cost and scalable approach to efficiently optimise outcomes for individuals with MDD in Pakistan.

**Disclosure of Interest:**

None Declared

